# Fluoride resistance in fibroblasts is conferred via reduced susceptibility to oxidative stress and apoptosis

**DOI:** 10.1002/2211-5463.12786

**Published:** 2020-01-27

**Authors:** Jing Ni, Zhe Zhong, Wu Zhang, Bin Liu, Rong Shu, Yiming Li

**Affiliations:** ^1^ Department of Periodontology Shanghai Key Laboratory of Stomatology & Shanghai Research Institute of Stomatology National Clinical Research Center of Stomatology Ninth People's Hospital Shanghai Jiao Tong University School of Medicine China; ^2^ Center for Dental Research Loma Linda University School of Dentistry CA USA

**Keywords:** apoptosis, fluoride resistance, mitochondria, oxidative stress, ROS

## Abstract

Chronic fluoride exposure from drinking water may result in endemic fluorosis. To better understand the mechanisms by which some people are resistant to fluorosis, here we investigated the effect of treatment with NaF (sodium fluoride) on production of reactive oxygen species (ROS), morphological changes in mitochondria, the mRNA expression of Fas ligand (Fas‐L), and the protein expression of cleaved caspase‐3 in regular L‐929 cells and fluoride‐resistant (FR) L‐929 cells. While morphological changes indicative of apoptosis and a network of fragmented mitochondria were observed in regular L‐929 cells after NaF treatment, there were no morphological changes in FR L‐929 cells after NaF treatment. Treatment with 10 mm NaF induced a significant difference in the production of ROS, triggered the expression of cleaved caspase‐3, and upregulated the mRNA expression of Fas‐L in regular L‐929 cells. However, there was no significant production of ROS in FR L‐929 cells. Additionally, cleaved caspase‐3 and upregulated Fas‐L were not detected in FR L‐929 cells. These results suggest that FR fibroblasts are resistant to oxidative stress and apoptosis induced by fluoride.

## Graphical abstract

To better understand the mechanisms by which some people are resistant to fluorosis, here we compared reactive oxygen species production, apoptosis, and morphological changes in mitochondria between regular and fluoride‐resistant (FR) cells after NaF treatment. We report that FR cells were resistant to oxidative stress and apoptosis induced by fluoride.

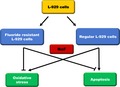

**Abbreviations**
Fas‐L: Fas ligand
FR: fluoride‐resistant
MTT: methyl thiazolyl tetrazolium
NaF: sodium fluoride
RPE: retinal pigment epithelial
ROS: reactive oxygen species

Fluoride is of great importance in the prevention and treatment of dental caries. Nonetheless, excessive chronic fluoride exposure from drinking water, contaminated dust, and fumes leads to endemic fluorosis, which mainly involves teeth and the skeleton 1, 2. The literature also indicates that 40% of the population in regions with high fluoride levels in the water is not affected by skeletal fluorosis 3. In addition, 5% of children do not suffer from dental fluorosis in high‐fluoride areas 4. These findings imply variations in individual susceptibility to the same level of fluoride exposure within the same population. This viewpoint is further reinforced by animal studies in which different mouse strains and their responses to fluoride were compared. The A/J mouse strain was highly susceptible to dental fluorosis, which progressed rapidly; however, the 129P3/J mouse strain was more resistant, manifesting mild dental fluorosis 5, 6. All these results show that individual susceptibility to fluoride exposure varies. However, the molecular mechanisms of fluoride resistance responsible for such variations are poorly understood.

Stimulation with NaF has been reported to increase the production of reactive oxygen species (ROS), causing oxidative stress 7, 8. Cellular apoptosis through the extrinsic or intrinsic apoptotic pathway is initiated by the overproduction of ROS 9, 10. The extrinsic pathway is activated by the interaction of the transmembrane death receptors TNF‐α or Fas with their ligands, which eventually leads to the activation of caspase‐3 11. In addition, oxidative stress causes damage to cellular components, especially the mitochondria, which are the site of ROS generation and also the target of oxidative stress‐induced damage 12.

We cultured fluoride‐resistant (FR) L‐929 cells developed from regular L‐929 cells (ATCC CCL‐1, Manassas, VA, USA). The method by which FR L‐929 cells were developed has been described in a previous study 13. In this study, we hypothesized that FR fibroblasts have self‐protective properties against fluoride‐induced oxidative stress and apoptosis. The aim of this study was to determine whether cellular fluoride resistance prevents apoptosis and oxidative stress through the inhibition of fluoride‐activated reactions involved in the production of ROS and the expression of caspase‐3 and Fas ligand (Fas‐L), which would partially explain the mechanisms of fluoride resistance.

## Materials and methods

### Assessment of cell morphological changes

Regular and FR L‐929 cells were exposed to 5 and 10 mm sodium fluoride (Sigma, St. Louis, MO, USA). After 12 h of stimulation, the cells were observed under a fluorescence microscopy manufactured by Thermo Fisher Scientific (EVOS, Boston, MA, USA) to evaluate morphological changes in the cells.

### Assessment of cell viability

The MTT assay was performed to evaluate cell viability. Regular and FR L‐929 cells were collected and dispensed at 3 × 10^5^ cells·100 μL^−1^ per well into 96‐well culture plates. After overnight incubation, the media were replaced with treatment media containing NaF at various concentrations (0.5, 1.0, 1.5, 2.0, 2.5, 3.0, 3.5, 4.0, and 4.5 mm), and medium without NaF served as the control. A 5 mg·mL^−1^ MTT solution prepared in medium was added to each of the cultured wells. After incubation for 4 h, 100 μL of 10% SDS in 0.01 N HCl was added. The O.D. of the plates at 570 nm was read using a microplate reader after incubation overnight.

### Immunofluorescence

To analyze apoptosis in regular and FR L‐929 cells after fluoride treatment, an immunofluorescence assay for cleaved caspase‐3 was performed. Both regular and FR L‐929 cells were treated with 10 mm NaF for 12 h. The cells were fixed with 4% paraformaldehyde at 4 °C for 15 min. Then, the cells were permeabilized with 1% Triton X‐100 and blocked with PBS containing 10% goat serum and 1% BSA for 30 min at 37 °C. The cells were stained with antibody against cleaved caspase‐3 (Cell Signaling Technology, Danvers, MA, USA) diluted 1 : 100 as the primary antibody overnight at 4 °C and HRP‐labeled goat anti‐rabbit diluted 1 : 200 as the secondary antibody (Santa Cruz Biotechnology, Dallas, TX, USA) at room temperature for 2 h. Nuclei were stained with DAPI.

### Real‐time quantitative RT‐PCR

Regular and FR L‐929 cells were exposed to 1 and 10 mm NaF for 12 h, and the RNeasy Mini Kit (Qiagen, Hilden, Germany) was then used to extract the total RNA. The quality and quantity of the RNA were analyzed with a NanoVue Plus (Biochrom US, Holliston, MA, USA). cDNA was synthesized from 500 ng of total RNA by using a QuantiTect Reverse Transcription Kit (Qiagen). The mRNA levels of Fas‐L were determined by qPCR (ViiA TM 7; Applied Biosystems, Thermo Fisher Scientific, Boston, MA, USA) using iTaq Universal SYBR Green Supermix (Bio‐Rad, Hercules, CA, USA). The forward primer for Fas‐L (5′ to 3′ direction) was TCCGTGAGTTCACCAACCAAA, and the reverse primer for Fas‐L (5′ to 3′ direction) was GGGGGTTCCCTGTTAAATGGG. The forward primer for GAPDH (5′ to 3′ direction) was AGGTCGGTGTGAACGGATTTG, and the reverse primer for GAPDH (5′ to 3′ direction) was GGGGTCGTTGATGGCAACA. The 2^−ddCt^ method was used to quantify target genes with GAPDH used as a reference gene.

### ROS measurement

To examine the effects of fluoride on the production of ROS in regular and FR L‐929 cells, 5(6)‐carboxy‐2′,7′‐dichlorofluorescein diacetate (CA‐DCF‐DA; Sigma‐Aldrich, St. Louis, MO, USA), a cytoplasmic ROS fluorescent indicator, was used. Both regular and FR L‐929 cells were treated with 5 and 10 mm NaF for 12 h; treatment with 0.5 mm H_2_O_2_ for 1 h was included as a positive control, and treatment with 10 mm NAc for 1 h was used as a negative control. Then, the cells were incubated with CA‐DCF‐DA (20 μm) for 30 min at 37 °C after stimulation. The cells were subsequently examined under a fluorescence microscope immediately and then collected in prewarmed PBS with 5% serum. The samples were subjected to flow cytometry with the FACSAria II cytometer (BD Biosciences, San Jose, CA, USA) at excitation and emission wavelengths of 470 and 529 nm, respectively.

### Detection of mitochondrial morphology

The MitoTracker probe (Invitrogen, Carlsbad, CA, USA) was used to label mitochondria. Both regular and FR L‐929 cells were treated with 10 mm NaF for 12 h. Prewarmed staining solution containing the MitoTracker probe (400 nm) was added. Then, the cells were incubated for 20 mins. After incubation, the cells were examined with a fluorescence microscope.

### Data statistical analysis

The results were calculated and are described as the mean ± SD. Data analysis was performed with spss (version 19.0, Chicago, IL, USA). Student's *t*‐test was adopted. Differences with a *P*‐value < 0.05 were considered significant.

## Results

### Morphological changes in regular and FR L‐929 cells and changes in cell viability following NaF treatment

**Figure 1:**
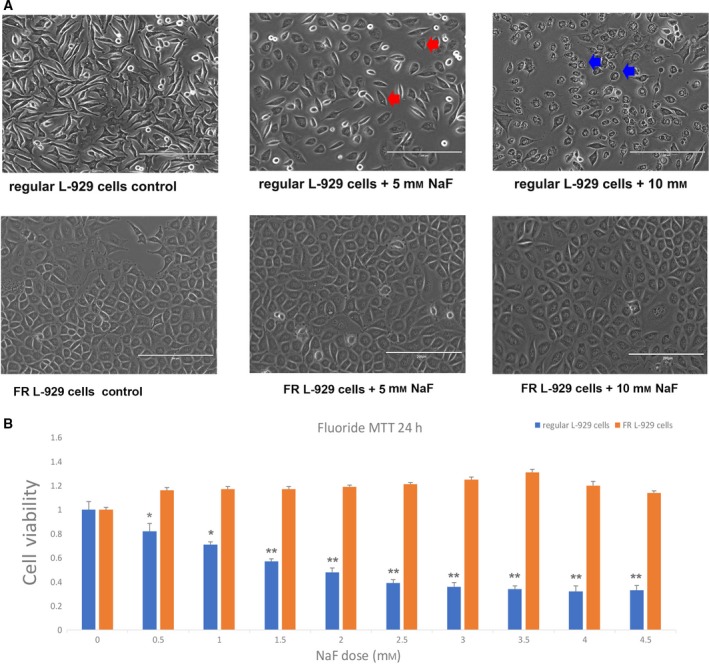
Changes in the morphology and cell viability of regular and FR L‐929 cells following stimulation with NaF. (A) The morphological difference between regular and FR L‐929 cells. The regular L‐929 cell has a spindle‐like shape, and the FR L‐929 cell is round. Morphological changes in regular L‐929 cells exposed to high concentrations of NaF were observed, while no morphological changes were observed in FR L‐929 cells exposed to NaF at the same concentration. Red arrows indicate cell shrinkage. Blue arrows indicate apoptotic bodies. Scale bars, 200 μm. (B) Both of the cell lines were treated with 0, 0.5, 1, 1.5, 2, 2.5, 3, 3.5, 4, and 4.5 mm NaF for 24 h. Cell viability was evaluated with the MTT assay. The viability of cells without NaF was set to 1. Each value (mean ± SD) represents the survival percentage relative to cells without NaF treatment. Asterisks indicate significant differences between the NaF‐stimulated group and the control group. Student's *t*‐test was adopted. (*P < 0.05, **P < 0.001).

Regular and FR L‐929 cells were stimulated with 5 and 10 mm NaF for 12 h. As shown in Fig. 1A, morphological differences between the FR and regular L‐929 cells were observed. Regular L‐929 cells had a spindle‐like shape, and FR L‐929 cells were round. In addition, a number of the morphological features of apoptosis were identified in regular L‐929 cells exposed to NaF. In regular cells treated with 5 mm NaF, cell shrinkage, which indicates the early stage of apoptosis, was obvious. In regular cells treated with 10 mm NaF, the cells became apoptotic bodies, indicating the late stage of apoptosis. However, no morphological changes were observed in FR L‐929 cells exposed to 5 or 10 mm NaF for 12 h. As illustrated in Fig. 1B, the viability of regular L‐929 cells was inhibited by NaF in a dose‐dependent manner, while the viability of FR L‐929 cells was not affected. The cell viability of regular L‐929 cells after treatment with NaF ranging from 0 to 4.5 mm for 24 h decreased from 100% to 31.77%. This decrease in cell viability was not observed in FR L‐929 cells exposed to NaF at the same concentrations. The 50% inhibitory concentration (IC_50_) for NaF‐induced growth inhibition in regular L‐929 cells was approximately 1.75 mm NaF at 24 h.

### Comparison of apoptosis in regular and FR L‐929 cells following treatment with NaF

**Figure 2:**
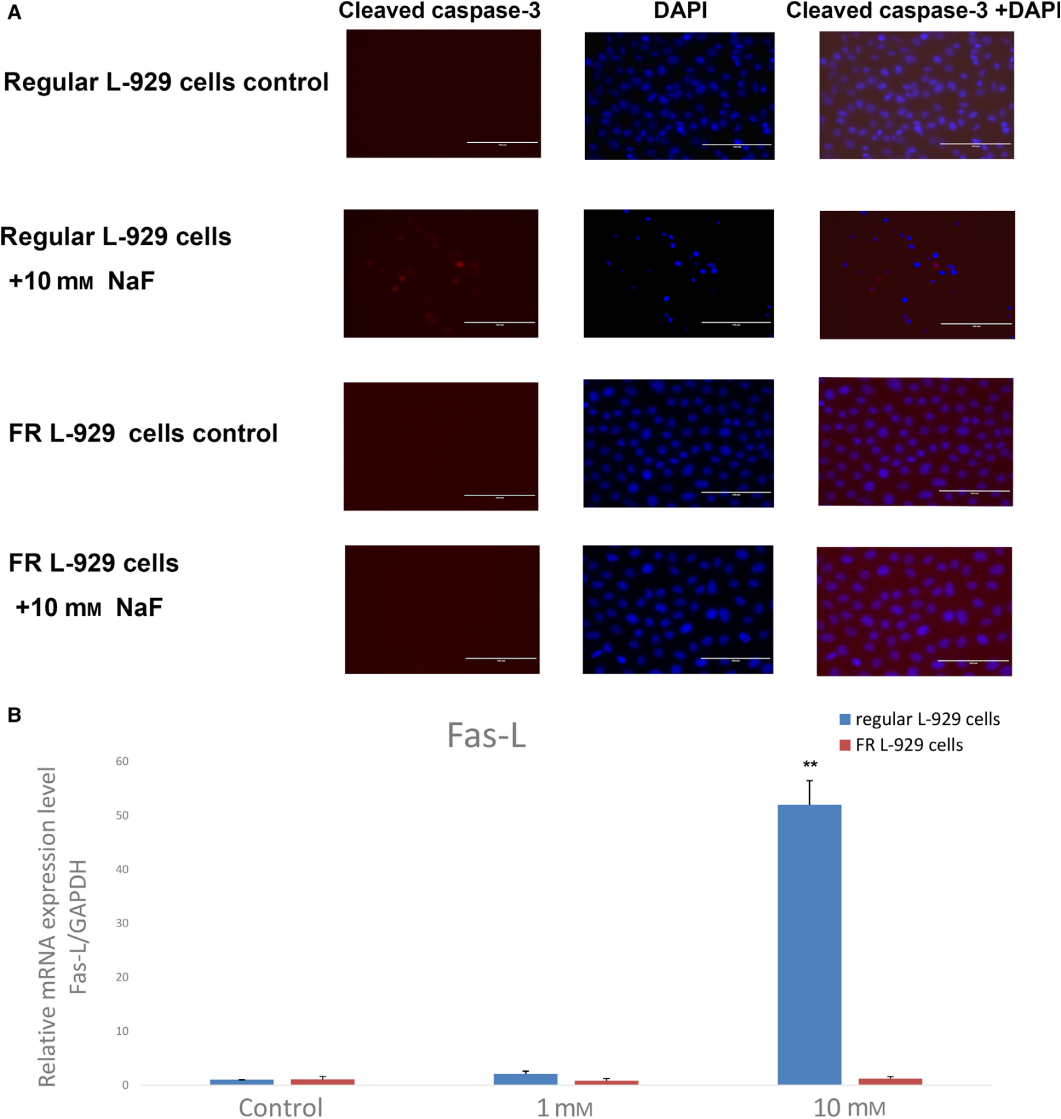
Comparison of apoptosis in regular and FR L‐929 cells following treatment with NaF. (A) Regular and FR L‐929 cells were stimulated with 10 mm fluoride for 12 h. Cleaved active caspase‐3 (red) was present in regular L‐929 cells after treatment; however, cellular staining was absent in FR L‐929 cells exposed to the same treatment conditions and untreated regular and FR L‐929 cells in the control groups. Changes in the nuclear morphology of regular L‐929 cells exposed to NaF were also observed, while FR L‐929 cells treated with NaF displayed normal, round nuclei. Scale bars, 100 μm. (B) Expression of Fas‐L mRNA in regular and FR L‐929 cells treated with 0, 1, and 10 mm NaF for 12 h was detected. The Fas‐L mRNA level was significantly increased in regular cells under 10 mm NaF stimulation compared to that in regular cells in the control group. However, there was no significant difference in Fas‐L mRNA levels in FR cells under 10 mm NaF stimulation compared to the FR cells in the control group. Values are the mean ± SD (*n* = 3). Student's *t*‐test was adopted (**P < 0.001).

To investigate whether apoptosis was activated in regular and FR L‐929 cells after fluoride treatment, an immunofluorescence experiment to detect cleaved caspase‐3 was carried out, and real‐time quantitative RT‐PCR was used to detect Fas‐L. Following 10 mm NaF treatment for 12 h, immunofluorescence analysis showed no detected cleaved caspase‐3 in FR L‐929 cells, but cleaved caspase‐3 was expressed in regular L‐929 cells. FR L‐929 cells exposed to fluoride displayed normal, round nuclei (Fig. 2A), while regular cells treated with fluoride exhibited nuclear condensation and fragmentation, which are features of apoptosis (Fig. 2A). These findings indicate that FR cells may be protected against apoptosis when exposed to high fluoride concentrations. The RT‐PCR results showed that the Fas‐L mRNA level was significantly higher in regular cells stimulated with 10 mm NaF; however, the Fas‐L mRNA level did not increase much in FR L‐929 cells treated with NaF at the same concentration (Fig. 2B). This finding suggests that the death receptor‐dependent apoptosis pathway is triggered in regular cells after treatment with a high fluoride concentration, while this is not the case in FR cells.

### Measurement of ROS generation in regular and FR L‐929 cells following treatment with NaF

**Figure 3:**
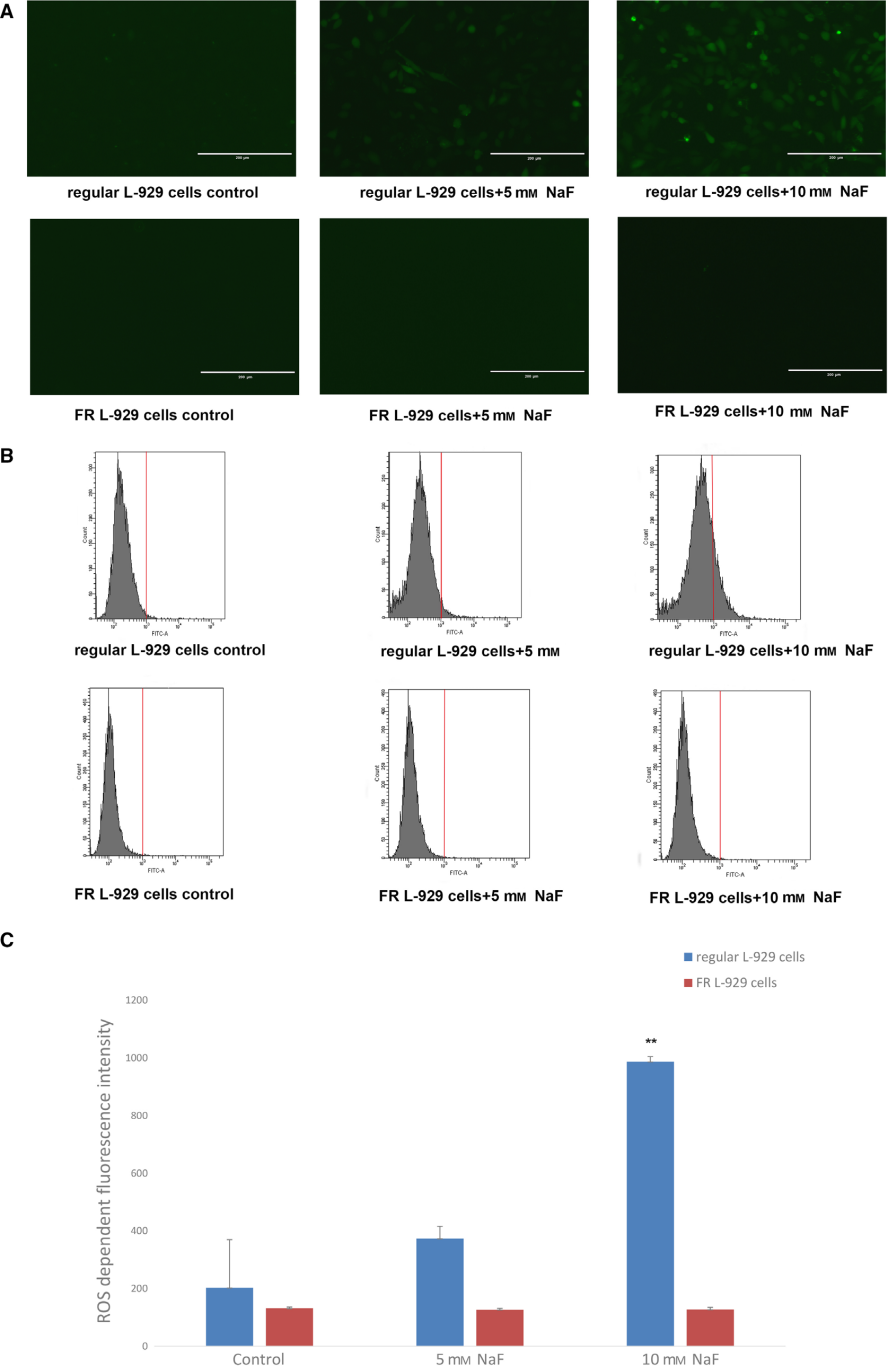
Measurement of ROS generation in regular and FR L‐929 cells following treatment with NaF. (A) ROS were detected by fluorescence microscopy. Regular L‐929 cells treated with fluoride showed increased fluorescence intensity, while the fluorescence intensity of FR L‐929 cells under the same treatment conditions was not different compared to that of FR cells in the control group. Scale bars, 200 μm. (B) Intracellular ROS levels were measured by flow cytometry analysis. Regular and FR L‐929 cells were stimulated with 5 and 10 mm NaF for 12 h. (C) Quantitative analysis of the mean fluorescence intensity of DCF. Treatment with 10 mm fluoride for 12 h significantly increased ROS generation in regular L‐929 cells. Values are the mean ± SD (*n* = 3). **P < 0.001, versus regular L‐929 cell control group. Student's *t*‐test was adopted.

The green fluorescence intensity of the probe used in this study represents the amount of ROS generated. In regular L‐929 cells treated with 10 mm fluoride, intracellular ROS levels were significantly enhanced in comparison with those in the regular cell control group. Meanwhile, the green fluorescence intensity in regular cells increased with increasing concentrations of NaF; however, fluoride treatment did not enhance green fluorescence intensity in the FR cells (Fig. 3).

### Morphologic changes in the mitochondria of regular and FR L‐929 cells exposed to fluoride

**Figure 4:**
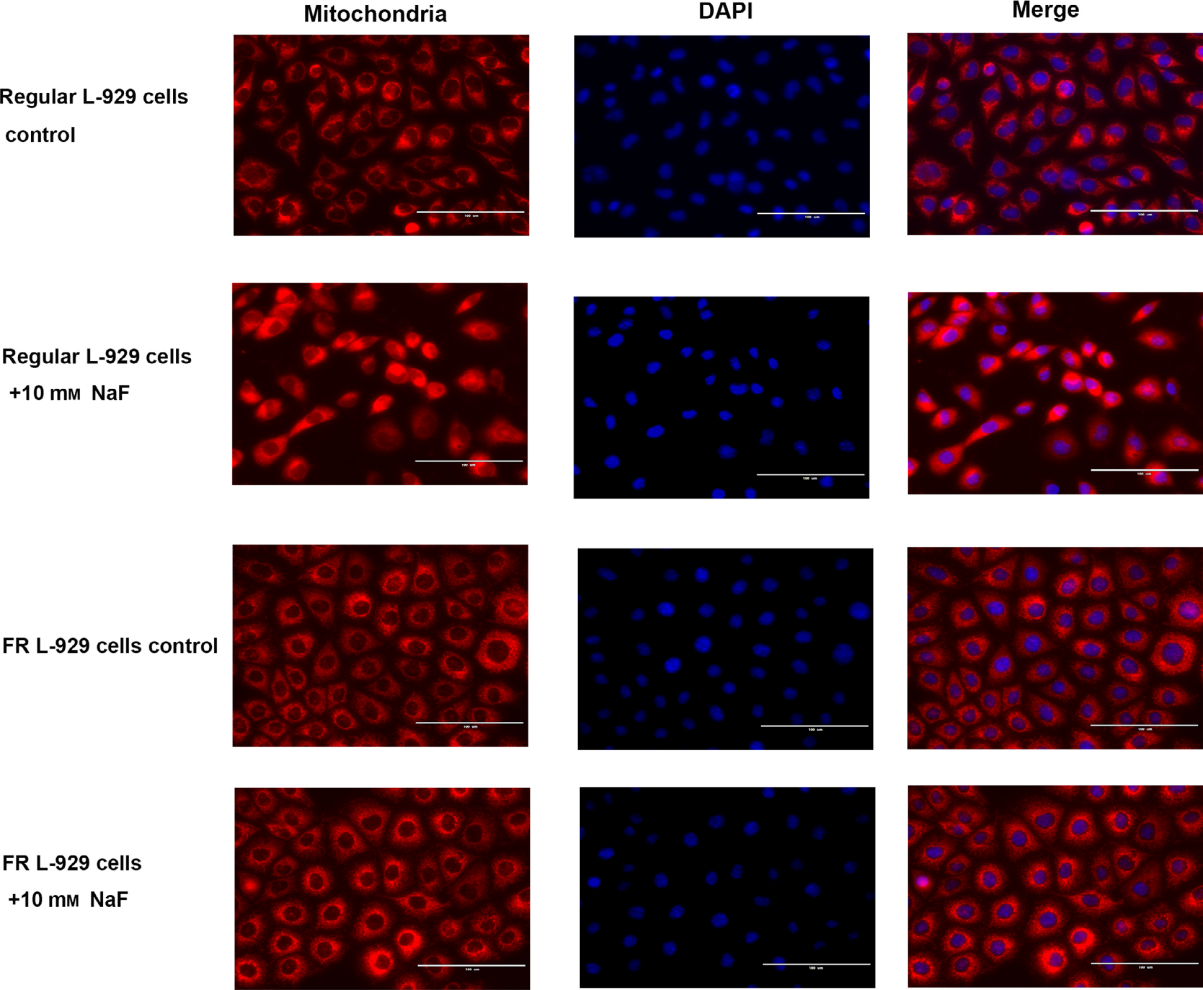
Morphologic changes in the mitochondria of regular and FR L‐929 cells exposed to fluoride. Regular and FR L‐929 cells were exposed to 10 mm fluoride for 12 h. The regular control group presented a normally shaped mitochondrial network, while regular cells treated with fluoride presented an abnormal fragmented mitochondrial network. Conversely, no mitochondrial morphological changes were observed in the FR cell control group and FR cells treated with fluoride. Furthermore, a difference in the mitochondrial morphology between regular and FR L‐929 cells was observed; the regular cells showed a slightly interconnected network of mitochondria, while the FR cells presented an intricate mitochondrial network that surrounded the nucleus. Scale bars, 100 μm.

The mitochondrial morphology of regular and FR L‐929 cells was analyzed with a fluorescence microscope. Both cell lines were induced with 10 mm NaF for 12 h and then stained with MitoTracker Red. As shown in Fig. 4, a difference in the mitochondrial morphology of regular and FR L‐929 cells was observed; the regular cells showed a slightly interconnected network of normally shaped mitochondria, while the FR cells presented an intricate network of mitochondria that surrounded the nucleus. After NaF treatment, an abnormal, fragmented mitochondrial network was observed in the regular cells. In contrast, no mitochondrial morphological changes were observed in the FR cells treated with fluoride (Fig. 4).

## Discussion

The ingestion of excessive fluoride can result in dental or skeletal fluorosis. A number of studies have indicated that individual susceptibility to fluoride exposure varies. However, most of the studies of this phenomenon have depended on epidemiological cohorts and animal experiments. To the best of our knowledge, the mechanisms of fluoride resistance at the cellular and molecular levels have not been investigated. The objective of our study was to determine whether fluoride resistant in cells can prevent oxidative stress and apoptosis induced by fluoride, which would partly explain the mechanisms of the fluoride resistance.

Apoptosis, also called programmed cell death, is the main cause of tissue injury and cell damage in endemic fluorosis. Many studies have reported that apoptosis is activated in several cell types, such as cementoblasts 14, ameloblasts 15, osteoblasts 16, odontoblasts 17, and gingival fibroblasts 18, after treatment with fluoride. In our study, the treatment of regular L‐929 cells with fluoride resulted in the cleavage of caspase‐3 and the upregulation of Fas‐L mRNA, but these effects did not occur in FR L‐929 cells exposed to the same treatment conditions, demonstrating that apoptosis was activated in regular cells after fluoride exposure via the extrinsic (death receptor) apoptotic pathway and that apoptosis was not initiated in FR L‐929 cells.

The finding that apoptosis was not initiated in FR L‐929 cells exposed to fluoride at a high concentration is of particular interest. Oxidative stress was reported to increase the expression of Fas and Fas ligand and activate apoptosis in murine intestinal epithelial cells 19. Tasharrofi N showed that miR‐374a, a negative regulator of the Fas death receptor, enhances human retinal pigment epithelial (RPE) cell survival and protects RPE cells against oxidative conditions 20. These findings indicate that oxidative stress is strongly related to activation of the extrinsic (death receptor) apoptotic pathway. Oxidative stress involves the excessive generation of ROS and damage to the mitochondria, which are the site of ROS generation and the target of oxidative stress‐induced damage 12. Our study shows that the generation of ROS was significantly increased in regular L‐929 cells treated with fluoride; however, the generation of ROS was not increased in FR L‐929 cells exposed to the same treatment conditions. Meanwhile, comparison of the mitochondrial morphology between regular and FR L‐929 cells after fluoride treatment demonstrated that the mitochondria in regular L‐929 cells treated with fluoride were damaged by the excessive production of ROS, but we did not find mitochondrial morphology changes in FR cells. All these results indicate that fluoride resistance in L‐929 cells can prevent oxidative stress, which partially explains the finding that apoptosis was not activated in FR L‐929 cells exposed to high fluoride concentrations.

Morphological differences in the mitochondria of regular and FR L‐929 cells in the control group were observed; regular L‐929 cells presented a slightly interconnected network of mitochondria, while FR L‐929 cells presented an intricate mitochondrial network around the nucleus. Mitochondria are very important in cellular bioenergetic and biosynthetic pathways 21. Effective communication between the nucleus and mitochondria helps cells adapt to stress 22. In FR L‐929 cells, the mitochondria form an intricate network around the nucleus, which may help mito‐nuclear cross‐communication and assist FR cells in adapting to the effects of fluoride; however, this hypothesis has yet to be proven.

## Conclusion

The present study first demonstrates that FR L‐929 fibroblasts have self‐protective properties against fluoride‐induced oxidative stress and apoptosis. Future studies examining mito‐nuclear cross‐communication in FR cells may help to elucidate the possible mechanisms that lead to individual susceptibility or resistance to the effects of fluoride.

## Conflict of interest

The authors declare no conflict of interest.

## Author contributions

YL, JN, and RS conceived and designed the project; JN, WZ, and ZZ performed the experiments; JN and BL analyzed the data; JN and ZZ wrote the paper; and YL made manuscript revisions. All authors read and approved the final manuscript.
